# Pathologic Features, Treatment, and Clinical Outcomes of Lacrimal Gland Cancer

**DOI:** 10.7759/cureus.44466

**Published:** 2023-08-31

**Authors:** Jerome M Karp, Alex J Gordon, Kenneth Hu, Irina Belinsky, Adam Jacobson, Zujun Li, Michael Persky, Mark Persky, Babak Givi, Moses M Tam

**Affiliations:** 1 Radiation Oncology, New York University (NYU) Langone Health, New York, USA; 2 Otolaryngology - Head and Neck Surgery, University of Pennsylvania, Philadelphia, USA; 3 Ophthalmology, New York University (NYU) Langone Health, New York, USA; 4 Otolaryngology - Head and Neck Surgery, New York University (NYU) Langone Health, New York, USA; 5 Hematology and Medical Oncology, New York University (NYU) Langone Health, New York, USA; 6 Head and Neck Service, Memorial Sloan Kettering Cancer Center, New York, USA

**Keywords:** adenoid cystic carcinoma, lacrimal sac, lacrimal duct, lacrimal gland, lacrimal gland cancer

## Abstract

Objectives: Lacrimal gland cancer is a rare malignancy with little data known about its pathologic characteristics or optimal management. We performed a large database analysis using the National Cancer Database (NCDB) to elucidate this unusual condition.

Methods: Patients with lacrimal gland cancer diagnosed between 2004 and 2018 were included in the analysis. Using available clinical data, we excluded all patients with histologies likely reflective of lacrimal sac or duct cancer, which are coded similarly to lacrimal gland cancer in the NCDB. Kaplan-Meier analysis was used to estimate overall survival (OS), and Cox proportional hazards models were used to indicate covariates associated with survival.

Results: A total of 440 cases of lacrimal gland cancer were included in the analysis, with a median follow-up of 52.9 months. The five-year OS for the entire cohort was 65.0%. Adenoid cystic carcinoma was the predominant histology (47.3%). Cox models showed that improved OS was associated with surgical resection (UVA: p < 0.001; MVA: p = 0.035). A detriment in OS was associated with increasing age, Charlson-Deyo score of 1, T4 stage, and positive margins and on UVA for adenocarcinoma and malignant mixed tumor histology.

Conclusion: Adenoid cystic carcinoma comprises the plurality of lacrimal gland cancers. About half of patients with lacrimal gland carcinoma will live beyond 10 years, underscoring the importance of reduced morbidity of treatment. Surgical management is associated with improved prognosis. Further study will elucidate the role of surgical excision and radiotherapy in lacrimal gland cancer.

## Introduction

Cancers of the lacrimal gland are extremely rare, with an incidence of only 1.3 cases per million per year [1]. The most common histology is adenoid cystic carcinoma, with adenocarcinoma, mucoepidermoid carcinoma, and malignant mixed tumor (carcinoma in pleomorphic adenoma) representing less common histologies [2].

Due to its rarity, the optimal management of lacrimal gland cancers has not been fully established. Traditional management has included surgical resection, either through orbital exenteration or globe-sparing surgery [2]. The addition of adjuvant radiation therapy is often considered for high-risk lesions, and the addition of neoadjuvant chemotherapy has also been suggested to confer benefit [3,4]. Three retrospective large-database analyses of lacrimal gland cancers have been published, two focusing on data from the surveillance, epidemiology, and end results (SEER) program [5,6], and another study, using the national cancer database (NCDB) [7]. However, a large fraction of cases analyzed in these studies represent histologies that are exceedingly rare in the lacrimal gland, including squamous cell carcinomas and transitional cell carcinomas. These tumors likely represent tumors from the lacrimal duct or lacrimal sac which have been miscoded [8]. Here, we present data from the NCDB on malignant lacrimal gland tumors, using published literature on lacrimal gland malignancy histologies to guide selection criteria, in order to elucidate pathologic characteristics, treatment patterns, and clinical outcomes for this rare entity.

This article was previously presented as a meeting abstract at the 2021 ASTRO Annual Meeting on October 26, 2021.

## Materials and methods

In this study, we queried the NCDB, a large, deidentified database produced as a joint collaboration between the American College of Surgeons' Commission on Cancer and the American Cancer Society, which captures 70% of all newly diagnosed malignancies in 49 US states and Puerto Rico, including a variety of care settings including academic hospitals and community cancer centers, among others. As the data were de-identified, this study was exempted from review by our Institutional Review Board. The NCDB was queried for cases of lacrimal gland cancer diagnosed between 2004 and 2018. Lacrimal gland cancer is coded in the NCDB by the ICD-O-3 site code C69.5. However, this code includes cancers of the lacrimal gland, lacrimal duct, and lacrimal sac. These sites vary significantly with regard to tumor histology. The NCDB includes a site-specific field, CS_SITESPECIFIC_FACTOR_25, which indicates the lacrimal apparatus subsite of the tumor. We excluded all tumors explicitly coded as situated in the lacrimal duct/sac; additionally, the newest cases included in the 2018 NCDB are also coded by lacrimal apparatus subsite using the SCHEMA_DISC_1 descriptor. We also excluded all squamous cell carcinomas and transitional cell carcinomas, both those without subsite codes and those explicitly coded as situated in the lacrimal gland, as these two histologies are considered extremely rare in the lacrimal gland, and likely reflect miscoding of malignancies in the lacrimal duct/sac [8]. Benign mixed tumors (pleomorphic adenoma) were excluded, and solitary fibrous tumors and fibrous histiocytomas were also excluded as all recorded cases of these tumors in the literature were benign [9]. We also excluded melanoma histology, and lymphoma histology is not included as it is assigned a different site code based on the ICD-O-3 classification. We excluded patients with less than six months of follow-up.

Cases were stratified by age (both as a continuous variable and as a categorical variable indicating age 60 years or greater vs. age less than 60), sex, race, year of diagnosis, facility type, histology, Charlson-Deyo comorbidity score, T stage, margin status, and presence of perineural invasion. We also stratified by patients who underwent surgical resection, radiation therapy, and chemotherapy. Overall survival (OS) was assessed using Kaplan-Meier statistics. Univariate and multivariate analyses were performed using the Cox proportional hazards model. All analysis was performed using the R language (r-project.org; the R Foundation, Vienna, Austria) and associated packages.

## Results

**Figure 1:**
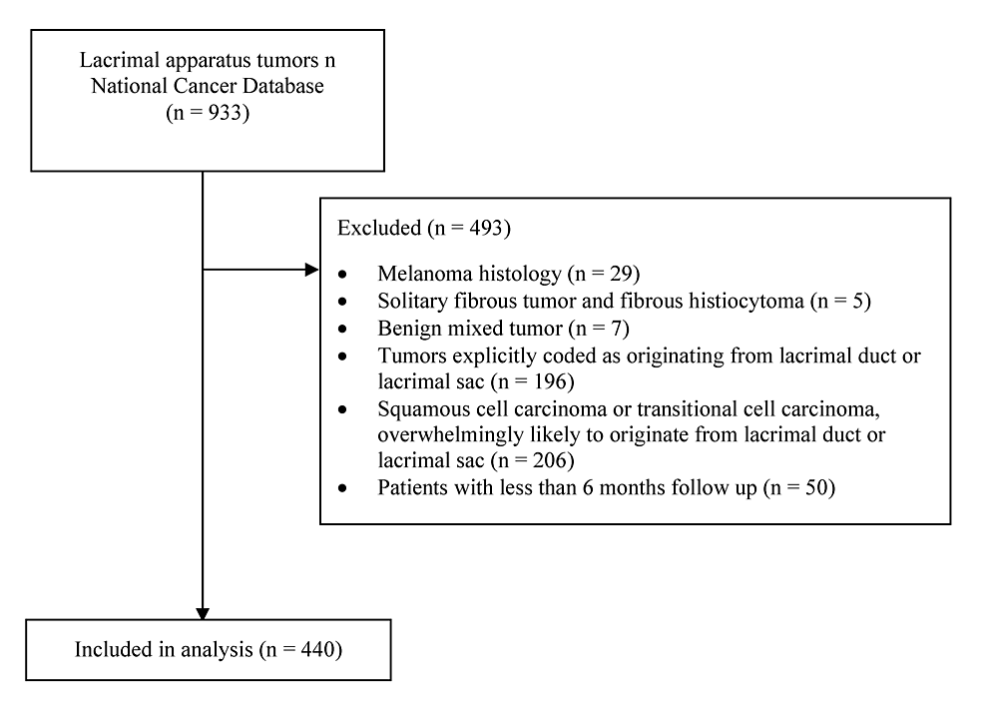
Inclusion diagram of the study

A total of 933 patients were included in the NCDB with lacrimal apparatus tumors. Of these, 450 cases were excluded as they were explicitly coded as lacrimal duct or sac tumors or they were tumors of histologies not typically found in the lacrimal gland as primary tumors, such as squamous cell carcinoma which may metastasize from a different primary. An additional 43 cases were excluded due to insufficient follow-up of less than six months. The remaining 440 cases were included in the analysis (Figure 1). Baseline characteristics of these patients are presented in Table 1.

**Table 1:** Baseline characteristics of the patients included in this study SD: standard deviation

n	440
Age, mean (SD)	55.60 (18.36)
Age >60	195 (44.3%)
Race	
White	312 (70.9%)
Asian/Pacific Islander	31 (7.0%)
Black	52 (11.8%)
Hispanic	33 (7.5%)
Other/unknown	12 (2.7%)
Sex	
Male	238 (54.1%)
Female	202 (45.9%)
Histology	
Adenoid cystic carcinoma	208 (47.3%)
Adenocarcinoma	99 (22.5%)
Malignant mixed tumor	14 (3.2%)
Mucoepidermoid carcinoma	44 (10.0%)
Other/unknown	75 (17.0%)
T stage	
T0-T1	48 (10.9%)
T2	57 (13.0%)
T3	18 (4.1%)
T4	105 (23.9%)
Unknown	212 (48.2%)
N stage	
N0	108 (24.5%)
N1	15 (3.4%)
Unknown	267 (72.0%)
Perineural invasion	
Present	100 (22.7%)
Absent	86 (19.5%)
Unknown	254 (57.7%)
Charlson-Deyo comorbidity score	
0	366 (83.2%)
1	64 (14.5%)
2+	10 (2.3%)
Facility type	
Community cancer program	61 (13.9%)
Academic/research program	238 (54.1%)
Integrated Network Cancer Program	50 (11.4%)
Unknown	91 (20.7%)
Underwent surgery	
Yes	343 (78.0%)
No	97 (22.0%)
Underwent radiotherapy	
Yes	218 (49.5%)
No	194 (44.1%)
Unknown	28 (6.4%)
Received chemotherapy	
Yes	112 (25.5%)
No	312 (70.9%)
Unknown	16 (3.6%)

The mean age of the patients included in this study was 55.6 years, with 44.3% of patients over the age of 60. Overall, 238 patients (54.1%) were male. The most common histology was adenoid cystic carcinoma, with 208 cases (47.3%). Other histologies included adenocarcinoma (99 cases, 22.5%), mucoepidermoid carcinoma (44 cases, 10.0%), and malignant mixed tumor (14 cases, 3.2%). Unfortunately, most patients did not have recorded T stage (48.2% not recorded) or perineural invasion status (57.7% not recorded). Details of surgery were available for the 343 patients who underwent surgery (Table 2). For 151 patients (44.0%), the surgery was coded as “enucleation,” although this is likely referring to orbital exenteration which is commonly employed for lacrimal gland carcinoma [2]. Ninety-five patients (27.7%) were noted to have simple/partial removal of the primary site or debulking surgery.

**Table 2:** Details of surgery among 294 patients who underwent surgery

Type of surgery	
Local tumor excision (not otherwise specified)	33 (11.2%)
Simple/partial removal of the primary site	83 (28.2%)
Total surgical removal of primary site/total enucleation	151 (51.4%)
Radical surgery	51 (17.3%)
Surgery stated to be debulking	12 (4.1%)
Not specified or unknown	13 (4.4%)
Surgical margins	
Positive	144 (32.7%)
Negative	171 (38.9%)
Unknown	125 (28.4%)

**Figure 2:**
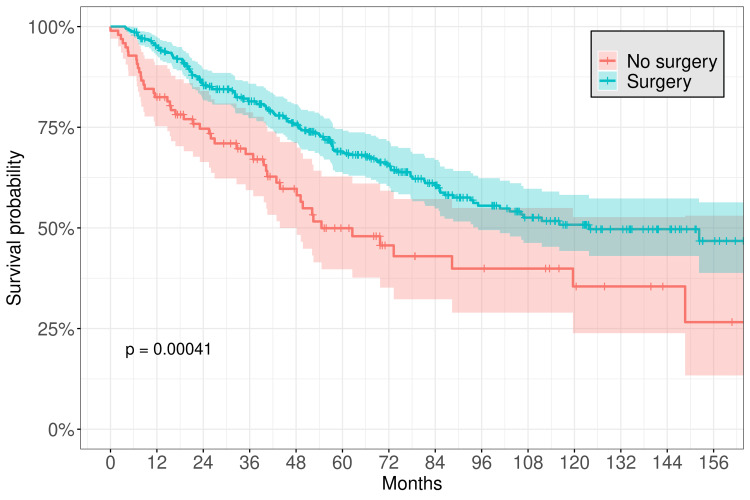
Kaplan-Meier plot for survival of patients associated with surgical resection

**Figure 3:**
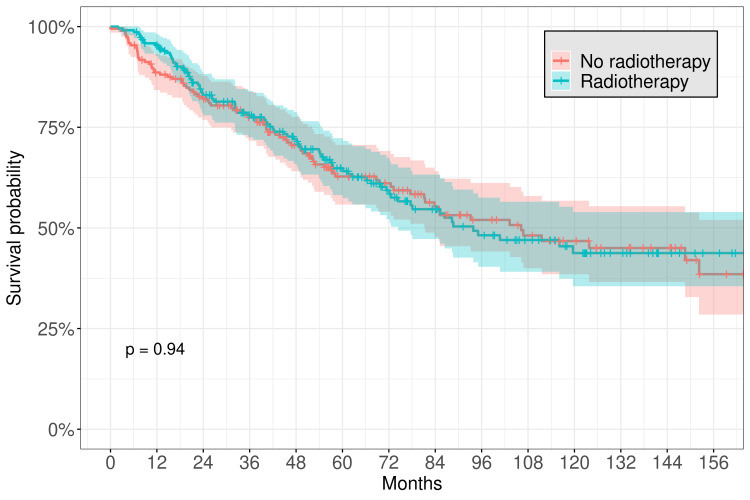
Kaplan-Meier plot for survival of patients associated with radiation therapy

The median follow-up time was 52.9 months. The five-year OS for the entire cohort was 65.0% (95% CI, 60.3-70.1), and the 10-year OS was 47.7% (95% CI, 41.8-54.3). The median OS was 107.0 months. By histology, adenoid cystic carcinoma had a median OS of 119.6 months, while adenocarcinoma had a median OS of 61.5 months, and malignant mixed tumor median OS was 34.2 months. Even more locally advanced tumors had prolonged survival: the median OS of T0-T2 tumors was 152 months, compared to 149 months for T3-T4 tumors. However, margin status did significantly impact OS, as patients with positive margins had a median survival of 85.3 months (95% CI 72.2-116), while patients with negative margins had a median survival of 182.4 months (95% CI 119.6-NR). Patients who underwent surgical resection had a significantly longer median survival time (123.8 months) compared to those who did not undergo surgical resection (54.6 months; log-rank statistic p < 0.001), with a five-year OS of 69.0% (95% CI 63.9-74.5) vs. 49.9% (95% CI 39.7-62.8) (Figure 2). However, there was no significant benefit associated with receiving radiation therapy, with a five-year OS for patients receiving radiation therapy of 64.8% (95% CI 58.1-72.3) vs. 62.8% (95% CI 55.8-70.6) for those who did not undergo radiation therapy (Figure 3). We also selected a subset of 211 high-risk patients who underwent surgical resection, including those with T3 or greater stage disease, presence of positive margins, or presence of perineural invasion. Even among this cohort, of which 121 patients (57.3%) were noted to have received radiation therapy, there was no increase in survival seen with radiation therapy (five-year OS 64.8% (95% CI 58.1-72.3) vs. 62.7, (55.8-70.6)). However, data regarding T stage, margin status, and perineural invasion was absent for most patients. Of note, more than half of all patients included in the analysis (54.1%) who received radiotherapy had either T3-T4 stage or positive margins, and this is likely an underestimate as T stage and margin status were often unrecorded. When selecting only patients with T3-T4 stage, there was also no difference in OS with radiation therapy (five-year OS 64.1% (95% CI 52.4-78.6) with radiotherapy vs. 62.9% (95% CI 49.8-79.6) without radiotherapy).

Univariate Cox regression (Table 3) showed a significant decrease in OS associated with increasing age (p < 0.001), adenocarcinoma (p = 0.001) or malignant mixed tumor (p = 0.026) histology, T4 stage (p = 0.027), positive margins (p = 0.009), and Charlson-Deyo score of 1 (p = 0.002). In contrast, undergoing surgical resection was associated with a significantly improved OS (HR 0.550, 95% CI 0.393-0.770, p < 0.001), as was treatment in an integrated network cancer program (p = 0.043). Multivariate Cox regression (Table 3) including age, histology, T stage, Charlson-Deyo score, surgical resection, and margin status as covariates showed that age (p < 0.001), Charlson-Deyo score of 1 (p = 0.031), T4 stage (p = 0.021), and positive margins (p = 0.022) were still associated with significantly worse survival, while surgical resection was associated with significant improvement in survival (p = 0.035).

**Table 3:** Results of the Cox proportional hazard model

	Univariate HR (95% CI)	p-value	Multivariate HR (95% CI)	p-value
Age (continuous)	1.033 (1.023-1.042)	<0.001	1.036 (1.022-1.050)	<0.001
Age > 60	2.207 (1.633-2.984)	<0.001		
Race				
White	Reference			
Asian/Pacific Islander	1.252 (0.721-2.176)	0.425		
Black	0.806 (0.479-1.356)	0.416		
Hispanic	1.129 (0.650-1.961)	0.667		
Sex				
Female	Reference			
Male	0.844 (0.626-1.137)	0.265		
Histology				
Adenoid cystic carcinoma	Reference		Reference	
Adenocarcinoma	1.893 (1.319-2.716)	0.001	1.359 (0.921-2.006)	0.122
Malignant mixed tumor	2.425 (1.114-5.279)	0.026	1.978 (0.890-4.393)	0.094
Mucoepidermoid carcinoma	0.874 (0.493-1.550)	0.646	0.994 (0.548-1.802)	0.984
T stage				
T0-T1	Reference		Reference	
T2	1.862 (0.842-4.118)	0.124	2.324 (1.016-5.317)	0.046
T3	1.149 (0.360-3.665)	0.815	1.147 (0.346-3.796)	0.823
T4	2.241 (1.122-4.479)	0.022	2.334 (1.135-4.792)	0.021
Positive margins	1.629 (1.128-2.354)	0.009	1.563 (1.066-2.291)	0.022
Perineural invasion	1.446 (0.834-2.509)	0.189		
Charlson-Deyo comorbidity score				
0	Reference		Reference	
1	1.806 (1.233-2.646)	0.002	1.553 (1.041-2.316)	0.031
2+	1.860 (0.760-4.555)	0.174	1.090 (0.431-2.757)	0.855
Facility type				
Community cancer program	Reference		Reference	
Academic/research program	0.700 (0.478-1.026)	0.068	1.003 (0.670-1.502)	0.989
Integrated network cancer program	0.557 (0.316-0.981)	0.043	0.706 (0.397-1.259)	0.238
Underwent surgery	0.550 (0.393-0.770)	<0.001	0.669 (0.461-0.972)	0.035
Underwent radiotherapy	0.987 (0.730-1.335)	0.932		
Received chemotherapy	0.918 (0.643-1.311)	0.639		

## Discussion

Because tumors of the lacrimal gland are extremely rare, information about their biological characteristics and outcomes of treatment are not well-established. Two studies of data from the SEER database have been published elucidating characteristics and patterns of care for lacrimal gland tumors [5,6]. However, both studies have raised questions regarding the data presented [8,10,11]. In particular, both reported high rates of squamous cell carcinoma. In one study, which included lymphomas (58.0%) in the presented data, 11.5% of cases were noted to be squamous cell carcinomas, compared to 13.4% adenoid cystic carcinoma [6]; in the other study which did not include lymphoma, 29.9% of cases were squamous cell carcinoma, compared to 32.1% adenoid cystic carcinoma [5]. These results are inconsistent with other published data regarding lacrimal gland cancers which suggest that adenoid cystic carcinoma represents the predominant histology, while squamous cell carcinoma is extremely rare [11,12]. Rather, these cases of squamous cell carcinoma, as well as the cases of transitional cell carcinoma presented in these studies, are highly likely to be tumors of the lacrimal duct or sac. In a more recent study querying the NCDB, 33.5% of cases were squamous cell carcinomas [7], again suggesting that virtually all these cases represented lacrimal duct or sac tumors. Unfortunately, the ICD-O-3 site code for all three subsites (the lacrimal gland, duct, and sac) is C69.5, increasing the difficulty of distinguishing these tumors and potentially altering the validity of outcome reporting in these large-database analyses.

In this study, we used the NCDB to report pathologic characteristics and outcomes for lacrimal gland cancers. Due to the above concerns of miscoding, we excluded all cases of squamous cell carcinoma and transitional cell carcinoma from analysis, as well as all cases that were explicitly coded as emanating from the lacrimal duct/sac using site-specific codes in the NCDB. We found that adenoid cystic carcinoma is the predominant histology, representing 47.3% of cases. Our analysis also shows good outcomes for patients with lacrimal gland cancers, with a median survival over eight years. Surgical resection is associated with a significant benefit toward survival, although it should be noted that patients who did not undergo surgery may have represented patients with comorbidities or with non-resectable disease, which would confound the benefit of surgical resection in lacrimal gland cancers. Radiation therapy was not found to be associated with improved survival, even in a cohort of high-risk patients with locally advanced cancers (T3-T4) or positive margins or perineural invasion. This latter finding was likely due to the fact that patients who underwent radiotherapy were those with worse disease characteristics that were not properly recorded in the database. For instance, data regarding T stage, perineural invasion, and margin status were not coded for a significant percentage of patients. A recent case series showed a significant association between perineural invasion of lacrimal gland carcinomas and local recurrence, suggesting that this data might be needed for appropriate risk stratification [13]. Adjuvant radiation therapy is generally recommended as the standard of care for all epithelial malignancies of the lacrimal gland and any cases of incomplete surgical resection [9], and is thought to be especially important for advanced adenoid cystic carcinoma, given its predilection for perineural invasion [9,14] and association with increased risk of distant metastases and worse survival [2,15]. Furthermore, two recent series showed high rates of local control associated with radiotherapy delivered after eye-preserving surgery [16,17]. Although data limitations preclude us from making definitive conclusions about the utility of radiotherapy in lacrimal gland cancers, our study suggests that following surgery, many patients will have positive margins, as about half of the patients for whom margin status was recorded were noted to have positive margins, which is in turn associated with worse prognosis, highlighting the potential value of neoadjuvant or adjuvant therapy. Indeed, given that patients who received radiation therapy had comparable outcomes to those who did not, and these patients generally included those with high-risk factors, it may be the case that radiation therapy can be used to compensate for these risk factors to result in similar outcomes compared to those patients without high-risk factors.

Our analysis is limited due to its nature as a retrospective study of a large database. We were unable to ascertain the specific surgical procedures that patients included in the analysis underwent. In some cases, patients were noted specifically to have “total enucleation” procedures (likely to refer to orbital exenteration) or specifically noted to have only partial removal of the primary site or debulking surgery; however, many patients were coded as having undergone “radical surgery,” and it is not possible to ascertain whether they underwent globe-sparing surgery or orbital exenteration. The small number of patients with definitive surgical details prevents us from reporting outcomes for orbital exenteration compared to globe-sparing surgery, which has been advocated for in recent reports [11,18,19]. Miscoding may also affect data with regard to histology. Although tumors explicitly coded as situated in the lacrimal duct or sac are excluded, the subsite is not recorded at all for many tumors. It was for this reason that we chose to exclude squamous cell and transitional cell carcinomas, but it is likely that some of the reported cases of adenoid cystic carcinoma and other histologies common to the lacrimal gland in fact were tumors of the lacrimal duct or sac. A further limitation of this study is that the NCDB only reports OS but not recurrence or local survival; as such, it is not possible to provide information about recurrence in this disease with improved OS in recent years.

## Conclusions

Our data confirm the predominance of adenoid cystic carcinoma histology in the lacrimal gland and confirm the key role of surgical resection in the management of lacrimal gland cancer. However, radiation therapy was not associated with a significant improvement in survival in this limited dataset, although these patients were often at higher risk of recurrence due to adverse prognostic factors such as higher T stage, positive margins, or perineural invasion. Radiation therapy may have a role in counteracting these high-risk features. Our data shows that nearly half of patients with lacrimal gland cancer survive for more than 10 years, underscoring the significant impact of treatment-related morbidity for this disease. More data will need to be collected to ascertain the role of adjuvant radiation therapy in these tumors and to assess the role of non-exenterative surgery in lacrimal gland cancer.

## References

[1] von Holstein SL, Therkildsen MH, Prause JU, Stenman G, Siersma VD, Heegaard S (2013). Lacrimal gland lesions in Denmark between 1974 and 2007. Acta Ophthalmol.

[2] Woo KI, Yeom A, Esmaeli B (2016). Management of lacrimal gland carcinoma: lessons from the literature in the past 40 years. Ophthalmic Plast Reconstr Surg.

[3] Meldrum ML, Tse DT, Benedetto P (1998). Neoadjuvant intracarotid chemotherapy for treatment of advanced adenocystic carcinoma of the lacrimal gland. Arch Ophthalmol.

[4] Tse DT, Benedetto P, Dubovy S, Schiffman JC, Feuer WJ (2006). Clinical analysis of the effect of intraarterial cytoreductive chemotherapy in the treatment of lacrimal gland adenoid cystic carcinoma. Am J Ophthalmol.

[5] Mallen-St Clair J, Arshi A, Tajudeen B, Abemayor E, St John M (2014). Epidemiology and treatment of lacrimal gland tumors: a population-based cohort analysis. JAMA Otolaryngol Head Neck Surg.

[6] Andreoli MT, Aakalu V, Setabutr P (2015). Epidemiological trends in malignant lacrimal gland tumors. Otolaryngol Head Neck Surg.

[7] Ashok Kumar P, Wang D, Huang D, Paulraj S, Sivapiragasam A (2022). Current trends in the management of epithelial lacrimal gland tumors: a retrospective national cancer database analysis. Cureus.

[8] Belliveau MJ (2015). Unusual lacrimal gland tumor epidemiology explained. JAMA Otolaryngol Head Neck Surg.

[9] Andreasen S, Esmaeli B, Holstein SL, Mikkelsen LH, Rasmussen PK, Heegaard S (2017). An update on tumors of the lacrimal gland. Asia Pac J Ophthalmol (Phila).

[10] Mallen-St Clair J, Arshi A, St John M (2015). Unusual lacrimal gland tumor epidemiology explained. JAMA Otolaryngol.

[11] Woo KI, Kim YD, Sa HS, Esmaeli B (2016). Current treatment of lacrimal gland carcinoma. Curr Opin Ophthalmol.

[12] Wright JE, Rose GE, Garner A (1992). Primary malignant neoplasms of the lacrimal gland. Br J Ophthalmol.

[13] Ford JR, Rubin ML, Frank SJ (2021). Prognostic factors for local recurrence and survival and impact of local treatments on survival in lacrimal gland carcinoma. Br J Ophthalmol.

[14] Ahmad SM, Esmaeli B, Williams M (2009). American Joint Committee on Cancer classification predicts outcome of patients with lacrimal gland adenoid cystic carcinoma. Ophthalmology.

[15] Esmaeli B, Ahmadi MA, Youssef A (2004). Outcomes in patients with adenoid cystic carcinoma of the lacrimal gland. Ophthalmic Plast Reconstr Surg.

[16] Woo KI, Sagiv O, Han J, Frank SJ, Kim YD, Esmaeli B (2018). Eye-preserving surgery followed by adjuvant radiotherapy for lacrimal gland carcinoma: outcomes in 37 patients. Ophthalmic Plast Reconstr Surg.

[17] Wolkow N, Jakobiec FA, Lee H, Sutula FC (2018). Long-term outcomes of globe-preserving surgery with proton beam radiation for adenoid cystic carcinoma of the lacrimal gland. Am J Ophthalmol.

[18] Esmaeli B, Yin VT, Hanna EY, Kies MS, William WN Jr, Bell D, Frank SJ (2016). Eye-sparing multidisciplinary approach for the management of lacrimal gland carcinoma. Head Neck.

[19] Lin YH, Huang SM, Yap WK (2020). Outcomes in patients with lacrimal gland carcinoma treated with definitive radiotherapy or eye-sparing surgery followed by adjuvant radiotherapy. Radiat Oncol.

